# Correlations between lung pneumonic lesions and serologic status for key respiratory pathogens in slaughtered pigs in northern Uganda

**DOI:** 10.1186/s40813-021-00233-y

**Published:** 2021-10-04

**Authors:** Peter Oba, Michel M. Dione, Barbara Wieland, Frank N. Mwiine, Joseph Erume

**Affiliations:** 1 International Livestock Research Institute, P. O. Box 24384, Kampala, Uganda; 2 International Livestock Research Institute, c/o AfricaRice Sahel Station, Dakar, Senegal; 3 Institute of Virology and Immunology (IVI), Mittelhäusern, Switzerland; 4 Department of Infectious Diseases and Pathobiology (DIP), Vetsuisse Faculty, University of Bern, Bern, Switzerland; 5 Department of Biomolecular Resources and Biolaboratory Sciences, College of Veterinary Medicine, Animal Resources and Biosecurity, Makerere University, P. O. Box 7062, Kampala, Uganda; 6 National Agricultural Research Organization, Abi Zonal Agricultural Research and Development Institute (Abi ZARDI), P. O. Box 219, Arua, Uganda

**Keywords:** Lira, Lesion scores, Lungs, Respiratory, Pigs, Pneumonia, Porcine, Uganda

## Abstract

**Background:**

A cross-sectional study of slaughtered pigs was conducted in Lira district, Uganda, to (1) determine the prevalence and severity of pneumonia and (2) establish relationships between pneumonia types and the serological status for key respiratory pathogens. Using enzyme-linked immunosorbent assays (ELISAs), sera were screened for antibodies against *Mycoplasma hyopneumoniae* (*M. hyo*), *Actinobacillus pleuropneumoniae* (*App*), porcine reproductive and respiratory syndrome virus (PRRSv) and porcine circovirus type 2 (PCV2). Postmortem, lungs were grossly scored for pneumonia types and pneumonic lesions. Pneumonia types were characterized as catarrhal purulent bronchopneumonia (CPBP), pleuropneumonia (PLP) and pleuritis. The percent of lung surface affected by pneumonia was determined by estimating the affected surface area of each lung lobe. Each lobe was assigned scores based on the approximate volume represented and the total percentage of lung surface affected obtained as a sum of individual lobe scores. *Metastrongylus spp.* helminth infection was determined by examining lungs for gross presence or absence. RStudio was used for data analysis and presentation. Wilcoxon rank sum tests were used to compare median pneumonia lesion scores and serostatus for each studied pathogen. An ordinal logistic regression model was fitted to evaluate the odds of multiple pneumonia, with pathogen serostatus and *Metastrongylus spp.* infection as predictors.

**Results:**

One hundred sixty-seven (n = 167) lungs were examined for pneumonic lesions. The prevalences of CPBP, PLP and pleuritis were 29.9% (95% CI 22.9–36.9), 74.2% (95% CI 67.5–80.9) and 17.3% (95% CI 22.4–36.3), respectively. The true prevalence of PCV2 was 9.7% (95% CI 4.5–16.8), that of PRRSv was 7.5% (95% CI 2.7–14.2), that of *M. hyo* was 11.5% (95% CI 7.2–18.0), that of *App* was 25.1% (95% CI 18.5–38.0), and that of *Metastrongylus spp.* was 29.3% (95% CI 22.9–36.6). The odds of multiple pneumonia forms increased in pigs with multiple pathogens (ORs 2.6, *p* = 0.01) and *Metastrongylus spp.* infestation (OR 2.5, *p* = 0.003), suggesting synergistic effects of coinfections in the induction of lesions.

**Conclusions:**

This study revealed a high prevalence and severity of pneumonic lesions in slaughtered pigs. It provides baseline information and evidence for the magnitude of pneumonia associated with the studied pathogens and justifies future studies on their potential economic impacts on Ugandan pigs.

## Background

Respiratory diseases contribute significant economic losses to swine producers worldwide through increased mortality, retarded growth rates, and reduced feed conversion and reproductive performance [1–3]. Other losses arise from additional costs of treatment [4], loss of potential revenue and vaccinations [5, 6]. Various infectious agents are associated with lung lesions at slaughter [7]. Among these agents, *Mycoplasma hyopneumoniae* (*M. hyo*), *Actinobacillus pleuropneumoniae* (*App*), porcine reproductive and respiratory syndrome virus (PRRSv), porcine circovirus type 2 (PCV2) and swine influenza viruses are the most important agents associated with gross pneumonic lesions in pigs [8, 9]. However, other bacterial or viral agents are known to contribute significant pneumonic lesions in pigs. For example, concurrent infections of *M. hyo* with other agents, such as PCV2 or PRRSv, have been found to increase the severity and duration of mycoplasma pneumonia [10].

To establish the contribution of respiratory pathogens to lung lesions, it is necessary to score lungs at slaughter. This enables monitoring of herd health [7, 11] and provides baseline information for future epidemiologic studies. One of the cost-effective methods for this purpose is abattoir surveys, as they provide a valuable source of data and information supporting herd health management decisions. Rapid gross visual and detailed lung scores are used to accurately assess the extent of pathological lesions associated with enzootic pneumonia in pigs due to the occurrence of distinct gross lesions [12, 13]. Serologic and clinical evidence provides useful information on the extent and severity of pulmonary lesions. In addition, it is important for monitoring growth, as pneumonic lesions (such as pleurisy) have been associated with growth retardation in pigs [14, 15].

In all types of production systems, pig growth is a key productivity indicator that is affected by respiratory disease in a herd, which in turn affects herd profitability. In Uganda, no information is available on the actual extent of pneumonia, its impact on growth and any associations of lung lesions with serologic or clinical profiles in pigs, as no studies have been previously conducted. Thus, the contribution of pneumonia to the overall economic performance of swine herds cannot be estimated, which hampers the design of effective interventions. In the selection of pathogens to be included, findings of previous prevalence and disease impact studies and DISCONTOOLS [16] were considered. Based on these considerations, PCV2, PRRSv, *M.hyo* and *App* were prioritized. The aims of this study were to (1) determine the prevalence and severity of pneumonic lesions in slaughtered pigs and (2) establish the relationships between lung pneumonic lesions and serologic status for selected respiratory pathogens detected in slaughtered pigs in Lira district, mid-northern Uganda.

## Materials and methods

### Study area and design

We conducted a cross-sectional slaughter slab survey in Lira district, mid-northern Uganda, from March to September 2019. Lira district is located at latitude 2° 14′ 59.64″ north and longitude 32° 53′ 59.46″ east. Pigs sampled in this study were sourced from within Lira district (~ 70%) and from the neighboring districts of Dokolo, Agago, Alebtong and Kole (~ 30%). Visits were made during early morning when slaughters were conducted and on days when the number of slaughters were known to be high.

### Sampling of slaughter slabs and pigs

The study was conducted in three purposely selected slaughter slabs in the district based on high daily slaughter capacity (range 8–20 pigs). These slabs represented approximately 60% of all pigs slaughtered in the district (*DVO, pers. comm*). The three (3) slaughter slabs were Teso Bar, Adekokwok and Amach market. In each slab, pigs brought for slaughter from different sources were randomly sampled (approx. 40%) on a given day. A list of all pigs brought for slaughter was made, and each was allocated a number (on a piece of paper) from which a simple random sample was drawn. Other characteristics of pigs (live weight using a measuring tape, body condition score (BCS) and sex) were recorded antemortem. The unit of measurement was the individual pig, and the outcome variable was the presence or absence of pneumonia.

### Determination of sample size

A recent study reported a seroprevalence of 20.9% for *M. hyo* in pigs in Lira district [17]. A review of lung scoring methods by Garcia-Morante et al. [18] showed that 80% of pigs infected with *M. hyo* had lung lesions. Using these figures, we estimate that the prevalence of pneumonia in pigs in Lira district was at least 16%. We assumed no clustering effect within a slab since pigs were purchased from different farms. To determine the prevalence of gross pneumonia, the required sample size for a 5% level of significance was derived from the equation [19]:

1 $$n = Z_{\alpha /2}^{2} pq/d^{2}$$

where Z_α/2_ is the standard normal deviation for α = 1.960; p = estimated proportion of pigs with gross pneumonia lesions = 0.16; q = the estimated proportion of pigs with no gross pneumonia = 1 − p = 0.84; and d, the effect size, is estimated to be 6% (d = 0.06). Using the above equation, a sample size of 144 pigs was computed.

### Blood sample collection

Antemortem blood samples were collected from pigs for serum preparation. Each pig was properly restrained as described in the ILRI Standard Operating Procedures (SOPs) manual, Sect. 2, part (c) & (d) [20]. Blood was collected from the jugular vein using a 21G, 1.5" needle into plain 5 mL BD® vacutainer tubes. The tubes were labeled and then placed in an ice box containing ice packs at 4 °C. After collection, the samples were delivered to the district veterinary laboratory, where they were left to stand at room temperature (20 °C) overnight. After 24 h, sera were harvested into 2 mL cryotubes (Sarstedt®, Germany), labeled and stored in a freezer at − 20 °C until use.

### Serologic analysis of sera

Serologic assays were performed at the College of Veterinary Medicine, Animal Resources and Biosecurity (CoVAB), Makerere University. Sera were screened using ELISA test kits for each of the four key pathogens using the protocols described by each manufacturer: *M. hyo* and *App*-ApxIV (IDEXX, Westbrook, Maine, USA) and PRRSv and PCV2 (Krishgen Biosystems, India). The results were computed as a sample-to-positive ratio (S/P) using the equation:

2 $${\text{S}}/{\text{P}} = \frac{{\left( {{\text{Sample OD}} - {\text{Average of negative control}}} \right)}}{{\left( {{\text{Average of positive control}} - {\text{Average of negative control}}} \right)}}$$

Cutoff sample-to-positive ratios (S/P%) for *M. hyo* were > 0.40 (positive) and < 0.30 (negative) and for *App* were ≥ 50% (positive) and < 40% (negative). PCV2 and PRRSv S/P cutoff ratios for positive and negative samples were ≥ 0.2 and < 0.2, respectively. Suspect samples were retested.

### Lung lesion scoring procedures

For the animals from which sera were collected, detailed scoring of lung lesions was conducted postmortem. To ensure the accuracy of data collected, records for each pig were entered into a sheet of paper at antemortem. The first author performed the lesion scoring while being assisted by a research assistant to record observations on an Excel-designed sheet. Lungs were isolated from the thoracic cavity, placed on a flat clean surface, palpated and scored for visible pneumonic and pleuritic lesions. Palpation for hardened areas of hepatization (pneumonia or pneumonia-like) was performed, and the percent involvement per lung lobe was recorded. Incisions onto the lung parenchyma using a surgical blade were made to identify and characterize any deep-seated lesions. The gross lung lesion scoring procedures for CPBP, PLP and pleuritis were performed as previously described [13, 21].

Lesions were classified as catarrhal purulent bronchopneumonia (CPBP), pleuropneumonia (PLP) or pleuritis [22]. CPBP is characterized by cranioventral consolidation, reddish-to-pink areas, and mucous or purulent exudate on the cut surfaces of the lung. PLP includes lesions that are typical of one or more consolidated focus, mainly in the caudal lobes, with red-to-dark areas with fibrinous pleuritis and hemorrhages and necrosis on the cut surface [13]. Pleuritis lesions were classified into 3 grades according to severity as described in previous studies [14, 21]. Using this method, grade 0 represents no pleuritis, grade 1 is where up to 5% of the lung surface is affected, and grade 2 is where > 5% of the lung surface is affected (adhesions between lung lobes or between lobes and the thoracic cavity, mediastinum or pericardium). The prevalence of pneumonia and the percent of lung tissue affected by pneumonia (proportion of lung surface visibly affected by pneumonia) were determined [23]. The percentage of pneumonia-affected lung area was based on the proportion of the lung surface that was abnormally firm and discolored [10, 24, 25]. To estimate the surface area grossly affected by pneumonia, the method of Halbur et al. [23] was used. Each lobe was assigned quantitative points based on the approximate volume represented by that lobe. A maximal score of 10 was possible for each: the right cranial, right middle, left cranial and left middle lobes. The accessory lobe was assigned 5 points. For the right and left caudal lobes, a score of 27.5 points (15 for dorsal and 12.5 for ventral parts) was possible, resulting in a maximum total of 100 points for the entire lung [23]. Score values for lung area affected by pneumonia ranged from 0 to 100%. For pleuritis scores, values ranged from 0 to 2. In brief, the following parameters were calculated:

3 $${\text{i}}.\quad {\text{Percent of lung surface affected by pneumonia }} = {\raise0.7ex\hbox{${\text{Total area affected}}$} \!\mathord{\left/ {\vphantom {{\text{Total area affected}} {100}}}\right.\kern-\nulldelimiterspace} \!\lower0.7ex\hbox{${100}$}}$$

4 $${\text{ii}}.{\text{ Percent prevalence of pneumonia}} = \frac{{\text{Number of lungs affected by pneumonia }}}{{\text{Total number of lungs examined}}} \times 100$$

### Scoring for lung helminth infestations

Gross helminth infestations (*Metastrongylus spp.*) were detected by examining the diaphragmatic lung lobes for wedge-shaped areas during the lung scoring procedures. Incisions were made on a grossly affected lung lobe with a surgical blade, or strips of one-centimeter tissue from the edge of diaphragmatic lung lobes were trimmed and squeezed to express adult worms (slender, 30–50 mm in length) from the bronchi [26]. Infestations were scored as present (code = 1) or absent (code = 0).

### Data analysis

Data were coded and entered into *Excel 16.0* (*Excel Corp, TX*). Missing data were omitted from the analysis. RStudio [27] was used to analyze and present summary statistics. Two response variables were defined: pneumonia type (CPBP, PLP or pleuritis), coded (yes = 1, no = 0) and total pneumonia scores (range 0–100%). The presence or absence of multiple pneumonia types in each lung (coded from 0 to 3) was used for ordinal logistic regression analysis. Multivariable logistic regression analysis was used to assess the odds of detecting pneumonia as an outcome variable with serostatus for respiratory pathogens as predictors. An ordered logistic regression model was fitted to evaluate the odds of detecting multiple pneumonia types as a dependent variable, with serostatus for different pathogens as predictors. Adjustment for *Metastrongylus spp.* as a potential confounder was made by checking for a change in the model coefficient at a 10% cutoff when it was excluded from the model. Wilcoxon rank sum tests were used to compare the median pneumonia lesion scores and serologic status for each pathogen at a significance level of α = 0.05.

## Results

In total, 167 pigs were sampled and examined from three selected slaughter slabs. Overall, more female pigs (55.7%) were sampled, of which 17.2% (n = 16) were pregnant. Live weights varied from 26 to 184 kg, while the age range was from 5 to 50 months. Table 1 below shows the summary statistics of the pigs sampled.

**Table 1:** Summary statistics of the pigs sampled

Slaughter slab	No. of pigs sampled, % (n)		Age (months) Mean ± SD	Live weight (kg) Mean ± SD
	Males	Females		
Teso bar	21.55 (36)	28.14 (47)	13.9 ± 9.1	58.7 ± 31.6
Adekokwok	2.4 (4)	5.4 (9)	13.6 ± 3.8	70.3 ± 16.8
Amach market	20.35 (34)	22.15 (37)	14.6 ± 6.8	68.6 ± 27.9
Totals	44.3 (74)	55.7 (93)	14.2 ± 8.0	63.6 ± 30.0

*SD* standard deviation

### Prevalence of studied respiratory pathogens

The true prevalence of PCV2 was 9.7% (95% CI 4.5–16.8), that of PRRSv was 7.5% (95% CI 2.7–14.2), that of *M. hyo* was 11.5% (95% CI 7.2–18.0), and that of *App* was 25.1% (95% CI 18.5–38.0). The prevalence of *Metastrongylus spp.* was 29.3% (95% CI 22.4–36.6).

### Prevalence of pneumonia

Overall, the prevalence of pneumonia was generally high in all slaughter slabs. The prevalence of gross pneumonia in the three slaughter slabs was highest in the Amach market (80.28%, 95% CI 70.68–89.90), followed by Teso Bar (79.5%, 95% CI 77.16–92.60) and then Adekokwok (69.2%, 95% CI 61.93–76.47).

### Prevalence of pneumonic lesions (CPBP, PLP and pleuritis) and other lesions observed

The prevalences of CPBP, PLP, pleuritis and lung abscesses were 29.9% (95% CI 22.9–36.9), 74.2% (95% CI 67.5–80.9), 17.3% (95% CI 11.6–23.2) and 2.39% (95% CI 0.052–4.73), respectively. Overall, PLP was the most prevalent pneumonic lesion observed. Approximately 30% of sampled pigs also had *Metastrongylus spp.* nematodes in the lungs.

### Relationships between total pneumonia scores and respiratory pathogen serologic status

**Fig. 1:**
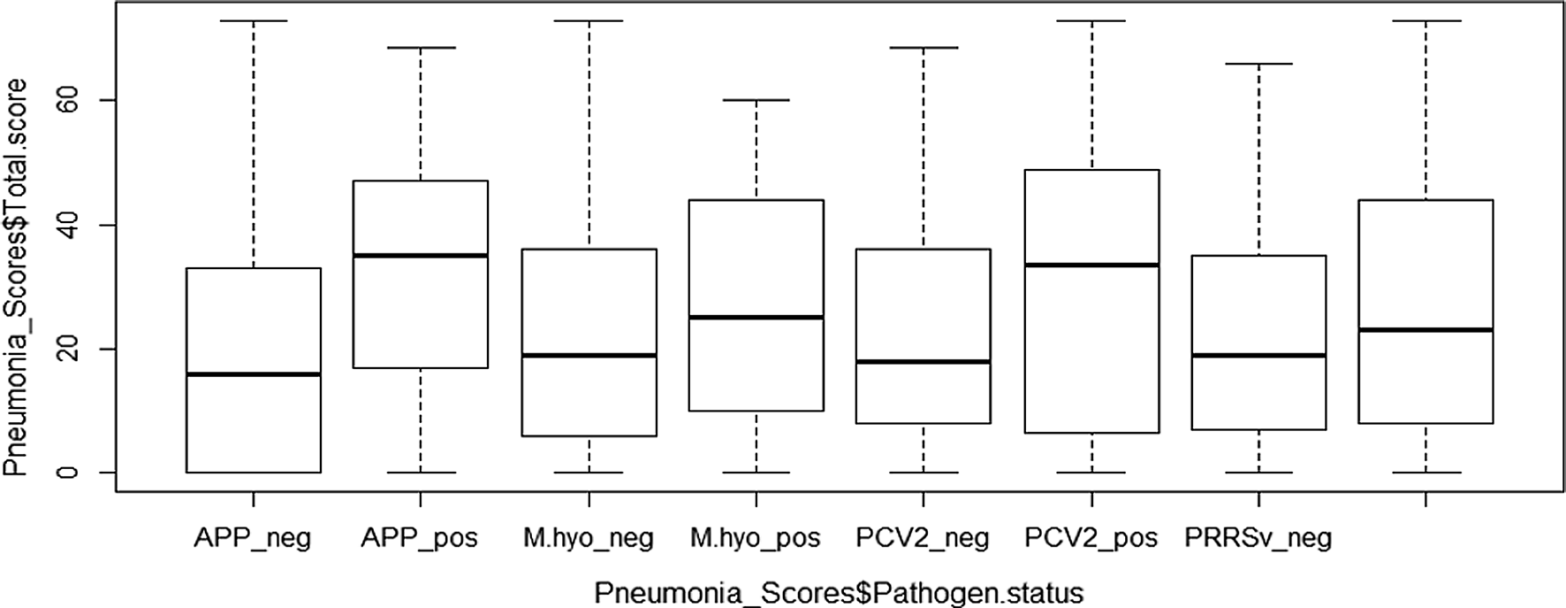
Boxplot of total lesion scores by pathogen serologic status

Figure 1 shows that the total median lesion scores for pigs that tested seropositive to each of the 4 pathogens were higher than those for pigs that tested seronegative. *App-*positive pigs showed significantly higher median lesion scores than *App*-negative pigs. Additionally, PCV2-positive pigs were found to have marginally higher total median lesion scores than PCV2-negative pigs. Figure 1 below highlights the summary statistics of the total median pneumonia scores by pathogen type.

### Relationship between pneumonia types and pathogen serologic status

Table 2 below summarizes the relationships between serologic status and median lesion scores (MLSs) of different forms of pneumonia. The Wilcoxon rank sum tests showed significant differences in CPBP and PLP scores between pigs that tested positive and those that tested negative for PRRSv, PCV2 and *M. hyo*. For pleuritis, a significant difference in median scores was observed between pigs that tested positive and those that tested negative for *M. hyo*. Table 2 below shows a summary of the results.

**Table 2:** Results of the Wilcoxon rank sum tests of median lung lesion scores by serologic status

Pneumonia type	Pathogen	Wilcoxon rank sum statistic (W)	Diff. median scores	Diff. 95% CI	*p* value
CPBP	PCV2	11,774	− 7.992e−05	− 7.813e−06 to − 6.574e−05	0.0006268***
PLP	PCV2	5678	− 0.999	− 1.000 to − 0.999	2.2e−16***
Pleuritis	PCV2	13,527	− 3.988e−05	− 1.382e−05 to 5.501e−05	0.4552
CPBP	PRRSv	11,523	− 1.841e−05	− 1.109e−05 to − 2.014e−05	0.000108***
PLP	PRRSv	5427.5	− 0.999	− 1.000 to − 0.999	2.2e−16***
Pleuritis	PRRSv	13,276	− 6.794e−05	− 7.875e−05 to 7.912e−06	0.2209
CPBP	*M. hyo*	11,189	−2.546e−05	− 1.593e−05 to − 2.734e−05	6.745e−06***
PLP	*M. hyo*	5093.5	− 0.999	− 1.000 to − 0.999	2.2e−16***
Pleuritis	*M. hyo*	12,942	− 3.381e−05	− 1.592e−05 to 5.807e−05	0.05723
CPBP	*App*	13,193	− 7.261e−05	− 4.332e−05 to 5.176e−05	0.2697
PLP	*App*	7097.5	− 0.999	− 0.999 to − 0.000	2.2e−16***
Pleuritis	*App*	14,946	5.213e−05	− 4.376e−05 to 2.098e−05	0.1074

^*^p < 0.05; **p < 0.01; *p* < 0.001***

### Ordinal logistic regression model of the effect of coinfections on multiple pneumonia occurrences

Table 4 below shows that the odds of scoring positive for multiple pneumonia types significantly increased in pigs with concurrent *Metastrongylus spp.* and respiratory infections.

Table 3 shows the effect of a single respiratory infection on the likelihood of detecting a given type of pneumonia. *Metastrongylus spp.* infestation of the lungs significantly increased the risks of occurrence of CPBP (OR = 2.29) and PLP (OR = 4.023). Infection with a single respiratory pathogen was not significantly associated with an increased risk of any form of pneumonia. However, coinfections with 2 respiratory pathogens and *Metastrongylus spp.* significantly increased the risks of occurrence of multiple pneumonia types (Table 4).

**Table 3:** Results of logistic regression models for pneumonia types and pathogen serologic status

Pneumonia type	Predictors	Estimate	Std error	OR	95% CI	z-value	Pr ( >|z|)
CPBP	Intercept	− 1.262	0.241	0.282	0.172–0.446	− 5.233	1.67e−07***
	*App*	0.540	0.384	1.717	0.799–3.638	1.405	0.1599
	*Met. spp.*	0.828	0.361	2.290	1.124–4.667	2.291	0.0219*
PLP	Intercept	0.645	0.202	1.906	1.291–2.859	3.194	0.00140**
	*Met. spp.*	1.392	0.515	4.023	1.583–2.398	2.704	0.00685**
	*M. hyo*	1.739	1.056	5.694	1.074–10.533	1.647	0.09956
Pleuritis	Intercept	− 1.8015	0.2647	0.165	0.095–0.269	− 6.805	1.01e−11***
	PCV2	0.4978	0.5286	1.645	0.543–4.452	0.942	0.346
	*App*	0.5633	0.4436	1.756	0.714–4.130	1.270	0.204

*Met. spp.* Metastrongylus spp, *ORs* odds ratios, *CI* conf. intervals

***p < 0.001; **p < 0.01; *p < 0.05

**Table 4:** Ordinal regression model of factors for the occurrence of multiple gross pneumonia types

Predictors	Coeff	Std error	ORs	95% CI	t-value	*p* value
Single infection	0.3446	0.365	1.411	0.690–2.898	0.943	0.345
Coinfection (2 pathogens)	0.9876	0.416	2.684	1.192–6.125	2.373	0.0176*
Coinfection (3 pathogens)	0.2313	1.037	1.260	0.155–10.131	0.223	0.823
*Metastrongylus spp.*	0.9526	0.330	2.592	1.364–4.993	2.883	0.0039**
Intercepts						
0|1	− 1.2504	0.250	–	–	− 4.989	0.0000***
1|2	1.2995	0.248	–	–	5.234	0.0000***
2|3	3.8065	0.444	–	–	8.563	0.0000***

*CI* conf. interval

*p < 0.05; **p < 0.01; ***p < 0.001

**Fig. 2:**
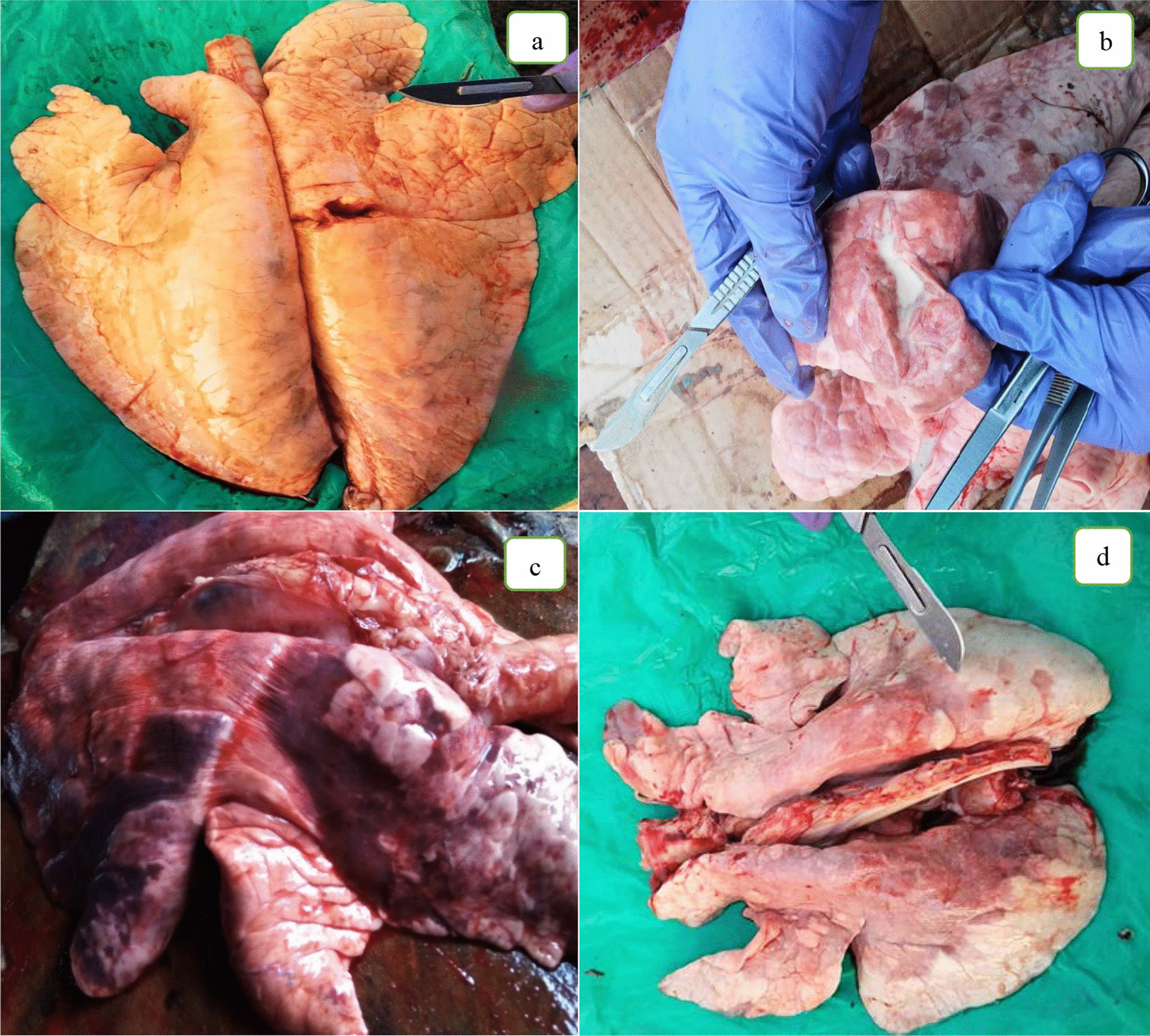

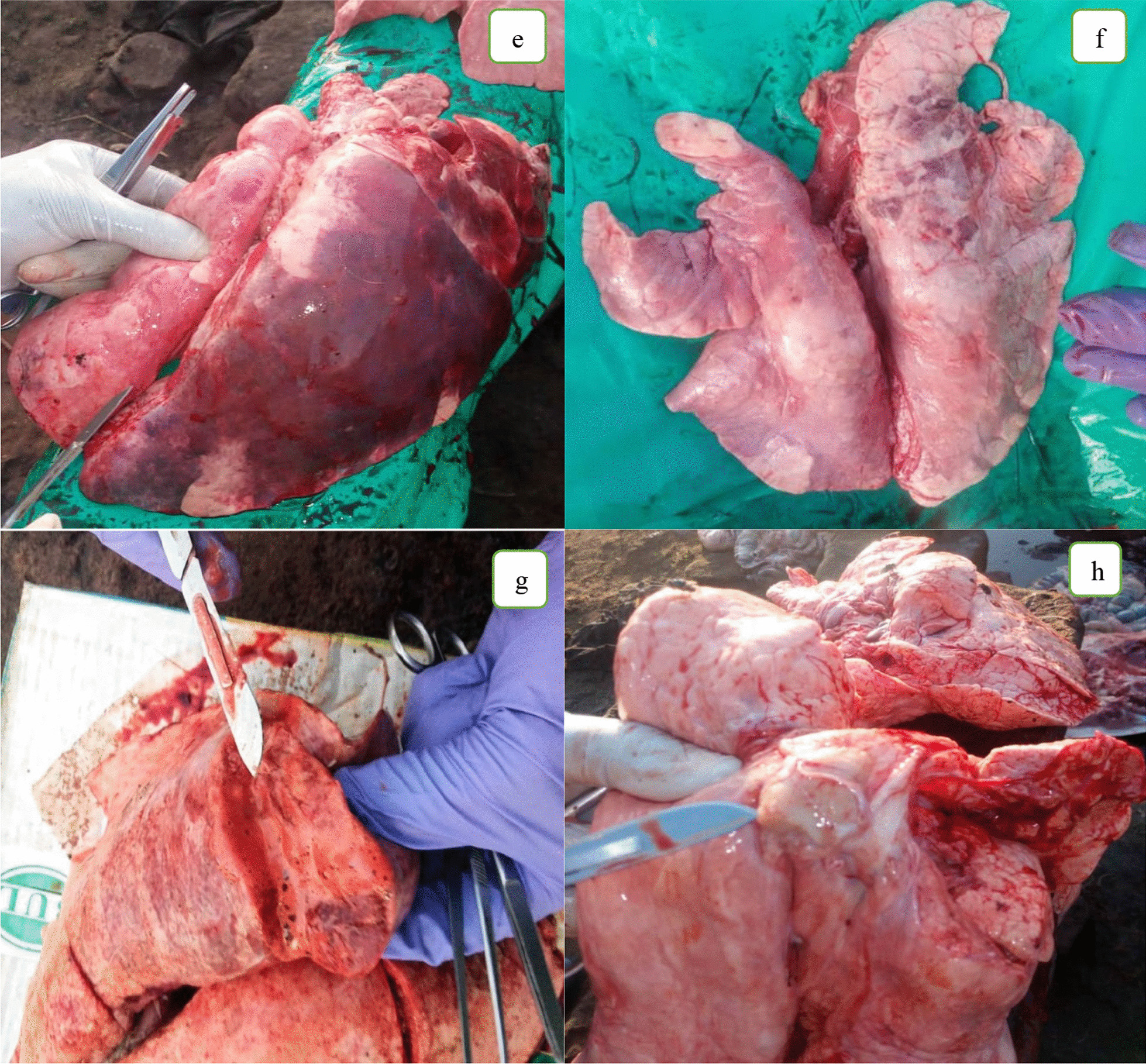
Pictures of normal lungs (**a**), lungs with purulent bronchopneumonia showing severe exudation (**b**); and cranioventral consolidation (**c**); lungs with diffuse interstitial pneumonia showing a rubbery texture (**d**); lungs with hemorrhagic bronchopneumonia showing failure to collapse (**e**) and pleuritis, showing attachments of lung lobes (**f**); hemorrhagic pleuropneumonia showing hemorrhages on cut surfaces of the lung (**g**), and a pulmonary abscess with thick caseous yellowish pus (**h**)

The Fig. 2a–h below present different gross forms of pneumonia observed in the lungs examined during the study.

## Discussion

This is the first study to document the magnitude of pneumonia prevalence and its relationship with respiratory pathogens in pigs in Uganda. This study revealed a high prevalence and severity of pneumonia in slaughtered pigs. The prevalence of pneumonic lesions (17.3–74.2%) in this study is comparable to that found in other studies, reported at 73.9% in Brazil [28]. Our findings compare favorably with studies in other countries that reported pneumonia prevalence ranging from 6 to 81% [11, 28–32]. Large variations in pneumonia and pleuritis (41–76% and 2–35%, respectively) have been reported in Brittany, France [14, 29]. In Makurdi, Benue State, Nigeria, it has been reported that 36.4% of sampled pigs had lung lesions [33]. In Ghana, it has been reported that only 5% of slaughtered pigs had pneumonia, although the form of pneumonia was not reported [34]. Differences in these studies likely reflect differences in scoring methods, production systems, hygiene and health status overall.

Of the studied respiratory pathogens, *Metastrongylus spp.* was found to be the most prevalent, followed by *App*. Our findings show that all the studied pathogens had low-to-moderate prevalence (from 7.5 to 25.1%) in the study area. The present study demonstrates significant associations between pneumonia type and serologic status for the studied pathogens. Significant differences in CPBP and PLP scores were observed between pigs that tested positive and those that tested negative for 3 pathogens: PRRSv, PCV2 and *M. hyo*. Pigs that tested seropositive to *App* showed a significant difference in median PLP scores between seropositive and seronegative pigs. Figure 1 shows that *App*-seropositive pigs had significantly higher total median scores than seronegative pigs. These associations between pathogen seropositivity and lesion scores suggest their probable role in lung pathology. This finding could explain the high prevalence of pneumonia observed in this study. Our findings agree with previous studies that reported *M. hyo* as being strongly associated with lung lesions [35] and pulmonary consolidation [36]. The finding that *App* was not significantly associated with pleuritis instead contrasts documented evidence that has shown *App* to be associated with fibrinous pleurisy [37]. This discrepancy could be due to infection with *App* serotypes of low virulence since the test used detects all serotypes of *App*.

The results of the logistic regression model showed that the odds of detecting CPBP and PLP increased in pigs infested with *Metastrongylus spp*. The finding that approximately one-third of the pigs sampled had *Metastrongylus spp.* nematodes, frequently observed in the tips of diaphragmatic lobes, agrees with a previous study, which found a high prevalence of GIT nematodes in Ugandan pigs [38]. GIT parasites such as *Ascaris suum* and *Metastrongylus spp.* are known to induce pulmonary tissue damage through their migratory larvae, increasing the susceptibility of pigs to various respiratory pathogens [26].

This study showed that the odds of detecting multiple pneumonia forms increased with coinfections. This result strengthens previous evidence that showed that coinfections tend to produce more severe disease than single infections. This finding corroborates previous studies that have documented synergistic or potentiating effects of coinfections between PRRSv and other pathogens [10] and PCV2 and other pathogens in the induction of respiratory disease [9, 39, 40]. The ability of *M. hyo* infection to potentiate and prolong PRRSv-induced pneumonia clinically and macroscopically has been documented [10].

Notwithstanding differences in the study design by [13], which sampled only heavy pigs (100 kg), our study sampled pigs of varying ages and live weights from predominantly small-scale production systems. The disease progression from acute to chronic as pigs grow older may also explain the differences in the lesion scores observed.

Apart from two studies in Nigeria and Ghana, there have been no other published studies in Africa (with comparable production systems) that have documented the magnitude of pneumonia prevalence in pigs. It is worth mentioning that in Uganda, no other published study or report on the magnitude of pneumonia prevalence in pigs exists. Thus, in the context of different pig production systems documented in Uganda [41], our findings can be extrapolated only to the swine population in northern Uganda, with similar husbandry systems. This study showed that a high proportion of pigs brought for slaughter in the region presented with a high prevalence and severity of pneumonic lesions and that the association between lesions and serologic status suggests a significant contribution of the studied pathogens to lung pathology. Due to variations in the pathogenicity of *M. hyo* and *App* serotypes, further studies are required to elucidate the identity and their role in the induction of lung pathology in pigs.

## Limitations of the study

The scoring methodology used in this study may have underestimated the actual magnitude of pneumonia since some pigs may have suffered early in life, and lesions could have resolved. In addition, due to the need to perform the scoring process quickly to match the slaughter speed, it is probable that some hidden lesions may have been missed. Since the ApxIV ELISA test detects all infections with *App* regardless of serotype, differences in virulence implies that not all lesions associated with *App* were correlated with positive serologic results.

## Conclusions

This is the first study to document associations of pneumonic lesions with serologic status for key swine respiratory pathogens in slaughtered pigs in Uganda. It revealed a high prevalence and severity of pneumonic lesions in slaughtered pigs in Lira district, and the association with respiratory pathogens suggests their potential contribution to lung pathology. The findings of this study establish critical baseline information for future studies on swine respiratory diseases. The high prevalence of pneumonic lesions justifies a need for future studies on the potential economic impacts of pneumonia on swine production and productivity in Uganda as a basis for designing future interventions.

## Data Availability

The datasets generated and analyzed during the current study are available at this link: https://data.ilri.org/portal/dataset/multipathogen-survey-and-risk-factors. We used the STROBE-VET checklist (https://strobevetstatement.files.wordpress.com/2016/09/strobe-vet-checklist.pdf) in the preparation of this manuscript.
